# Clinical Observations and the Anatomical Basis of Blindness After Facial Hyaluronic Acid Injection

**DOI:** 10.1007/s00266-019-01374-w

**Published:** 2020-07-01

**Authors:** Lei Zhang, Lei Pan, Hong Xu, Sheng Yan, Yi Sun, Woffles T. L. Wu, Sufan Wu

**Affiliations:** 1 Department of Plastic and Reconstructive Surgery, Zhejiang Provincial People’s Hospital, People’s Hospital of Hangzhou Medical College, Hangzhou, 310014 China; 2 Woffles Wu Aesthetic Surgery, Camden Medical Centre, 1 Orchard Boulevard, Suite 09-02, Singapore, 248649 Singapore

**Keywords:** Blindness, Hyaluronic acid, Hyaluronidase, Facial fillers, Visual impairment, Ophthalmic artery embolism, Retrobulbar injection

## Abstract

**Background:**

Blindness or visual loss is the most serious complication resulting from facial hyaluronic acid (HA) injection. In this study, three recent clinical cases were analyzed, and the relevant anatomy of cadavers was evaluated to investigate the mechanism behind visual impairment due to HA injection.

**Methods:**

Three patients with different extents of visual loss after HA injection were studied. Ophthalmic testing and corresponding treatments were performed, and the clinical progress was observed. In addition, thirty-six fresh Asian cadaver hemifaces were anatomized to investigate the morphology of the ophthalmic artery and its branches. The minimum dose of HA for central retinal artery embolism was calculated based on the ophthalmic arterial volumes of cadavers.

**Results:**

Visual impairment was more severe in central retinal artery occlusion and combined intraocular branch occlusion than in posterior ciliary artery occlusion. During follow-up, no improvement was observed in terms of visual impairment. Cadaver study reconfirmed that the ophthalmic artery included facial and intraocular branches. The ophthalmic arterial volumes running from the supraorbital artery and supratrochlear artery to the central retinal artery were 0.083 cm^3^ and 0.089 cm^3^, respectively.

**Conclusions:**

The severity of blindness caused by HA injection may be associated with the occlusion site. Our clinical observations indicate that conventional treatments, such as retrobulbar hyaluronidase injection, are insufficient to relieve visual impairment. Injecting as little as 0.08 ml of HA into the facial branch is enough to cause central retinal artery embolism. Limiting the volume per injection could represent a simple prophylactic strategy.

**Level of Evidence V:**

This journal requires that authors assign a level of evidence to each article. For a full description of these Evidence-Based Medicine ratings, please refer to the Table of Contents or the online Instructions to Authors www.springer.com/00266.

## Introduction

Hyaluronic acid (HA) is widely used as a facial filler because of it has long-lasting and less immunogenic properties than those observed for other temporary fillers and in addition, can be removed by dissolution with hyaluronidase [1]. All injectable facial fillers can cause complications, such as asymmetry, redness and even skin necrosis, and late complications can include granuloma and dyspigmentation [2, 3]. Blindness is the most serious complication resulting from injection of facial fillers [4–6]. Retinal photoreceptor cells, including cone cells and rod cells, have very short ischemic tolerance times, and irreversible ischemic necrosis is thought to occur within 90 min if the embolic occlusion is not removed [7, 8]. Treatment of embolic occlusion should be considered an emergency due to the limited ischemic tolerance of the retina [6].

Previous anatomical studies have shown that embolism of injectable material into branches of the ophthalmic artery, particularly the supratrochlear and supraorbital arteries, may lead to ophthalmic artery embolism and subsequent visual loss [9–11]. Relevant anatomical studies on the ophthalmic artery and its branches have already been reported [12–14]. Nevertheless, the minimum dose of HA that will produce central retinal artery embolism needs to be further studied [15, 16].

The extent of blindness or vision loss due to HA injection can differ among patients. Most cosmetic physicians are not familiar with the anatomy of the periorbital vasculature and that significant communications may exist between the facial artery and its branches (external carotid artery system) with the ophthalmic artery (internal carotid artery system) via the supraorbital, supratrochlear, anterior ethmoidal and superficial temporal arteries. To date, there is no widely recognized effective treatment to relieve this complication. Retrobulbar injection of hyaluronidase has been suggested as the first-line method to treat this catastrophic situation [17]. Retrobulbar injection has been proposed based on the phenomenon that extravascular injection of hyaluronidase can relieve cutaneous necrosis caused by intravascular HA embolism, and this may suggest this approach is potentially effective for visual impairment [18]. There is still a lack of consensus regarding the effectiveness of this treatment [19–22].

In this study, the authors reviewed three cases with different types of visual impairment caused by facial HA injection in which two patients were treated with emergency retrobulbar injection of hyaluronidase. The relevant anatomy was also investigated to calculate the minimum dose of HA that can cause central retinal artery embolism from facial branch injection.

## Methods and Materials

### Clinical Observation

From 2016 to 2018, three patients suffered from visual impairment after facial HA injection at private clinics and were transferred to the authors’ hospital for emergency treatment. All of the patients were women with ages ranging from 23 to 37 years old. Patient No. 1 suffered from complete visual field loss of the left eye and ophthalmodynia after HA injection in the nasal region. Patient No. 2 suffered from a visual defect on the upper side of the right eye, diplopia (right eye, oblique, 15 degrees) and limitation of medial motion after HA injection in the glabellar region. Patient No. 3 suffered from complete visual field loss and ptosis of the right eye after HA injection in the frontal and glabellar region.

All three patients suffered from visual impairment immediately after HA injection, and all three patients received initial hyaluronidase injection in the sites of the filler injections by their cosmetic physicians in their private offices (Table 1). Patient No. 1 was transferred to the hospital after 2 h and did not receive retrobulbar hyaluronidase injection. Patients No. 2 and No. 3 were transferred to the hospital after 4 h and 1 h, respectively, and received retrobulbar injections consisting of a total of 1500 IU of hyaluronidase to each affected eye by an ophthalmologist. The other treatments administered in these three patients are also described (Table 1).

**Table 1:** Disease profiles and treatment courses for patients

	Patient 1	Patient 2	Patient 3
Gender	Female	Female	Female
Age (years)	23	37	35
Filler injection region	Nasal region	Glabellar region	Frontal and glabellar region
HA injected volume	2.0 ml	1.5 ml	1.2 ml
Diseased eyes	Left eye	Right eye	Right eye
Extent of vision loss	Complete visual field loss	Partial visual field loss	Complete visual field loss
Time to vision loss	Immediately	Immediately	Immediately
Blepharoptosis	Severe	Mild	Severe
Retinal pale edema	(+)	(−)	(+)
Optic disk edema	(+)	(+)	(+)
Macular cherry red	(+)	(−)	(+)
Light perception existed	(−)	(+)	(−)
FFA	No fluorescence filling	Fluorescence hyperplasia	Fluorescence leakage
ICGA	Partial absent	Partial absent	Partial absent
Diagnose	CRAO, PCAO	PCAO	RRAO, PCAO
Time to hyaluronidase (Skin injection)	Immediately	Immediately	Immediately
Time to hyaluronidase (Retrobulbar injection)	2 h(−)	4 h	1 h
Retrobulbar injection	(−)	(+) (300 U/ml, total 1500 U)	(+) (300 U/ml, total 1500 U)
Glucocorticoid	(+)	(+)	(+)
Anticoagulant	(+)	(+)	(+)
Hyperbaric oxygen	(+)	(+)	(+)
Vision loss	No improved	No improved	No improved
Follow-up (months)	6	6	12

Ocular angiography FFA is to evaluate the central retina artery occlusion and ocular angiography ICGA is to evaluate the posterior ciliary artery occlusion

*CRAO* central retina artery occlusion; *PCAO* posterior ciliary artery occlusion; *BRAO* branch retinal artery occlusion

**Fig. 1:**
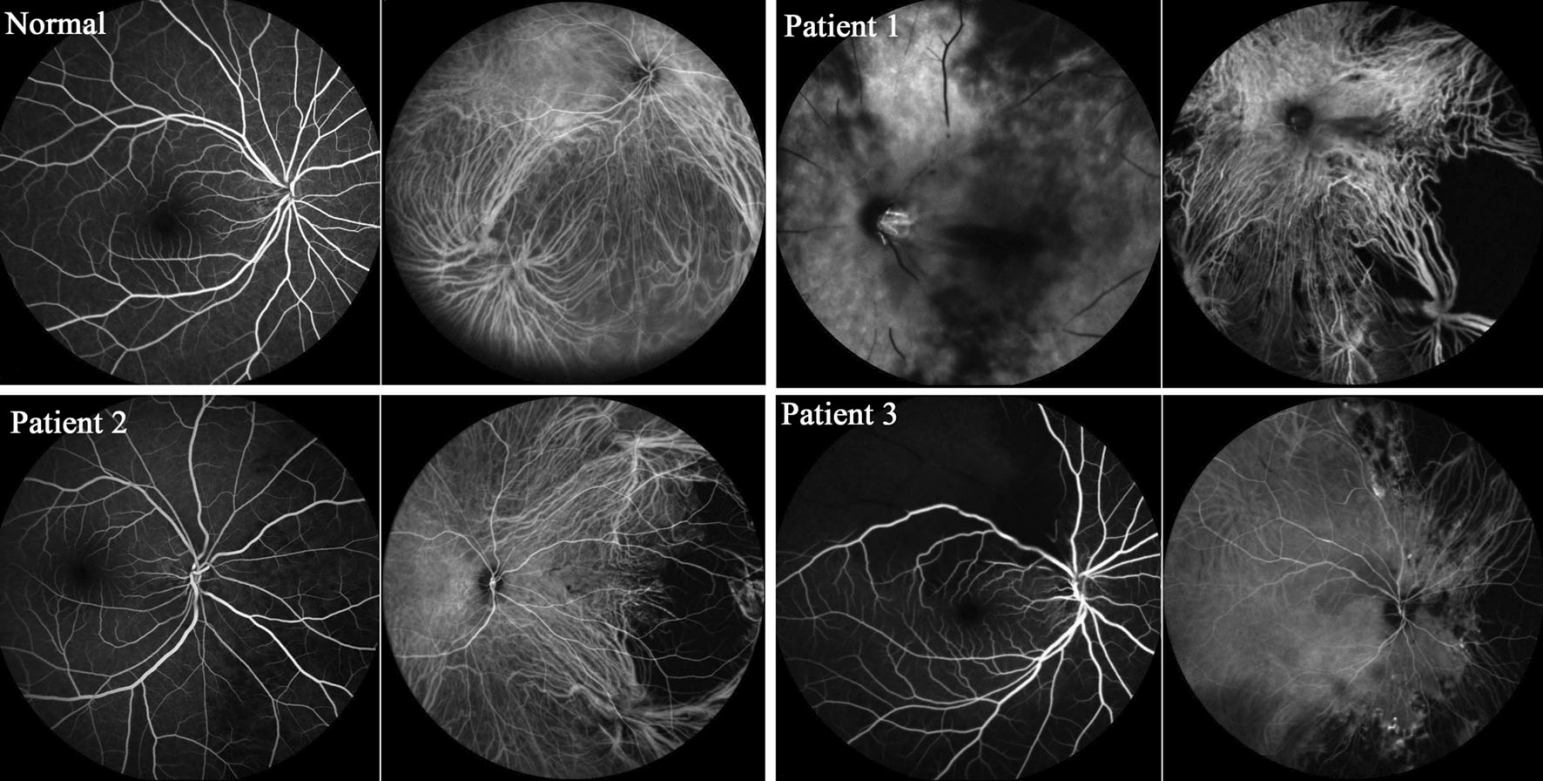
Ocular angiography of the patients. Patient No. 1: FFA: no fluorescein filling in the central retinal artery. ICGA: background fluorescence partially absent in the choroid. Patient No. 2: FFA: fluorescein filling of the central retinal artery. ICGA: background fluorescence partially absent in the choroid. Patient No. 3: FFA: fluorescein filling of the superior temporal retinal artery delayed and reversed. ICGA: fluorescein leakage; choroid filling partially absent on the nasal side

Fundus examination and ocular angiography were performed on these patients. A fluorescein angiography (FFA) examination was performed to evaluate central retinal artery occlusion, and the fluorescein filled the central retinal artery and its branches in the normal eye. Indocyanine green angiography (ICGA) examination was performed to evaluate posterior ciliary artery occlusion, and the choroid was full of fluorescence in the normal eye (Fig. 1).

Despite these emergency remedial injections of hyaluronidase, there was no improvement in the visual acuity of these patients and all three remained blind in the affected eye.

### Cadaver Study of the Ophthalmic Artery

Eighteen fresh Asian cadaver head specimens with red latex-filled arteries were anatomized. The subcutaneous vessels were observed after the skin was dissected and lifted. After the forehead skin and frontalis muscle were dissected, the supratrochlear artery, supraorbital artery and dorsal nasal artery were exposed. The points at which these arteries passed through the orbital margin were marked by ligatures with nylon sutures. The eyeball connected to the ophthalmic artery system and the optic nerve was excised from the orbital socket. All connective tissues were carefully dissected to keep the arteries, optic nerve and eyeball intact.

The lengths and external diameters of the supratrochlear and supraorbital arteries (from the orbital margin to their points of origin at the ophthalmic artery) and the central retinal artery (from the insertion point at the optic nerve to the eyeball) were measured to calculate the minimum volume of HA that would cause central retinal artery embolism and consequent visual impairment. The optic nerve was dissected to observe the central retinal artery, and a histological examination was also performed.

## Results

### Clinical Observation

Patient No. 1: Fundus examination: Left eye: pale retina, retinal edema, optic disk edema, central retinal artery occlusion, varicose veins, macular cherry red spot, no light perception. Right eye: no abnormalities. Ocular angiography after retrobulbar injection of hyaluronidase: Left eye: FFA: no fluorescence in the central retinal artery. ICGA: background fluorescence partially absent in the choroid, macular edema. Right eye: no abnormalities. Diagnosis: central retinal artery occlusion of the left eye, partial posterior ciliary artery occlusion of the left eye (Fig. 1).

Patient No. 2: Fundus examination: Left eye: no abnormalities. Right eye: optic disk hyperemia/edema, flat retina, light macular color, diffuse reflection. Visual field examination: defect of the upper right eye. Ocular angiography after retrobulbar injection of hyaluronidase. Left eye: no abnormalities. Right eye: FFA: fluorescein filling of the central retinal artery. ICGA: background fluorescence partially absent in the choroid, late fluorescein leakage. Diagnosis: partial posterior ciliary artery occlusion of the right eye, ischemic neuropathy of the right eye (Fig. 1).

Patient No. 3: Fundus examination: Left eye: no abnormalities. Right eye: pale retina, retinal edema with involvement of the macular area, retinal artery stenosis, whitening, pale optic disk, optic disk edema, no light perception. Ocular angiography after retrobulbar injection of hyaluronidase: Left eye: no abnormalities. Right eye: FFA: fluorescein filling of the superior temporal retinal artery delayed and reversed fluorescein leakage. ICGA: background fluorescence partially absent on the nasal side of the choroid; not improved in the middle and late stage. Diagnosis: superior temporal retinal artery occlusion of the right eye, partial posterior ciliary artery occlusion, macular ischemia/edema, ischemic neuropathy of the right eye (Fig. 1).

Patients No. 2 and No. 3 had received retrobulbar and glabellar region injections of hyaluronidase after the accident, whereas patient No. 1 did not receive retrobulbar injection but received nasal region injection of hyaluronidase. All three patients were treated with glucocorticoids, anticoagulants, vasodilators and hyperbaric oxygen in our hospital. The cutaneous ischemic necrosis improved in these patients, but none of the patients showed any improvement in vision after 6–12 months of follow-up (Table 1).

### Cadaver Study of the Ophthalmic Artery

**Fig. 2:**
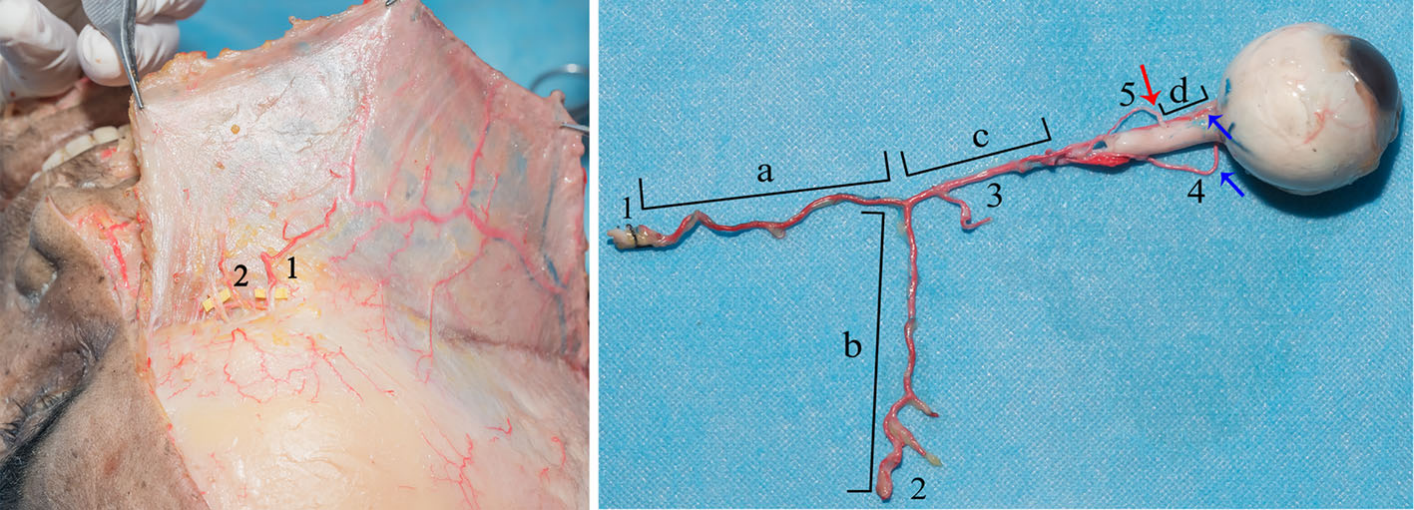
Branches of the ophthalmic artery in the forehead. The galea aponeurotica was elevated to expose the arteries. The supraorbital artery (1), supratrochlear artery (2) and periosteal branches ascend from the orbital margin, and the supraorbital artery has an anastomosis with the superficial temporal artery. The ophthalmic artery and its branches were carefully dissected and removed with the eyeball from the orbital cavity. The supraorbital artery (1) and the supratrochlear artery (2) are terminal branches of the ophthalmic artery (3). The lengths of the supraorbital artery (a) and the supratrochlear artery (b) and the distance from the origin point of the two branches to the origin point of the central retinal artery (c) were measured. The posterior ciliary arteries (4) reach the sclera of the eyeball. The central retinal artery (5) inserts perpendicularly into the optic nerve (red arrow) at a specific distance from the eyeball (d) and the posterior ciliary arteries have several collateral circulations (blue arrow)

In the forehead, the supraorbital, supratrochlear and dorsal nasal arteries arising from the orbital margin are located beneath the frontalis muscle and followed as they gradually inserted into the muscle; these represent the facial branches of the ophthalmic artery. In addition, several small branches arose from the orbital margin and extended up to the periosteum. The external diameters of the supratrochlear artery and supraorbital artery were approximately 1 mm each, and that of the ophthalmic artery was approximately 2 mm (Fig. 2).

The lengths of the supraorbital artery and supratrochlear artery from the orbital margin to the trunk of the ophthalmic artery were 4.2 ± 1.66 cm (Fig. 2a) and 4.6 ± 1.57 cm (Fig. 2b), respectively. The distance of the ophthalmic artery from the origin point of the facial branches to the origin point of the central retinal artery was 1.9 ± 0.47 cm (Fig. 2c). Based on these data, the arterial volumes from the orbital margin of the supraorbital artery and the supratrochlear artery to the branch point of the central retinal artery were calculated as 0.083 ± 0.027 cm^3^ and 0.089 ± 0.034 cm^3^, respectively (*a*/*b* × 3.14 × 0.05 × 0.05 + *c* × 3.14 × 0.1 × 0.1).

**Fig. 3:**
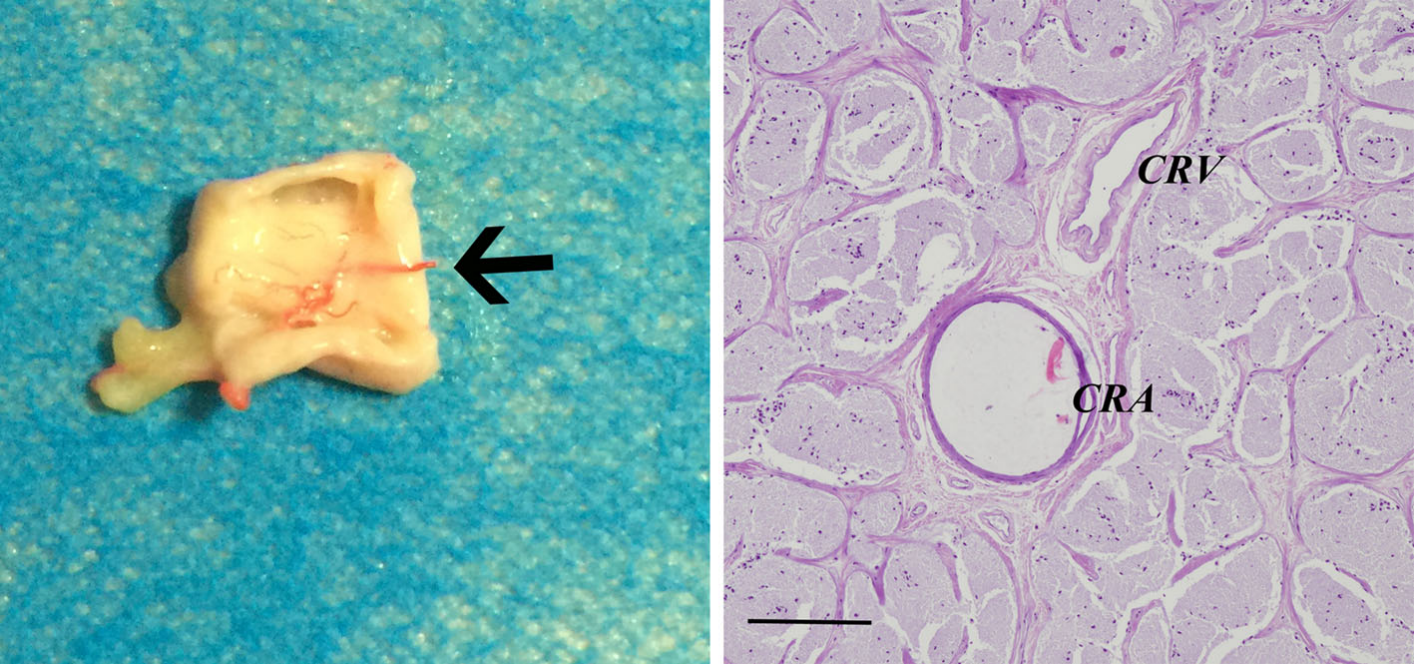
Cadaver anatomy of the central retinal artery. The central retinal artery is the only artery that inserts into the optic nerve (black arrow) and is surrounded by the optic nerve. Histological examination of the optic nerve revealed that the central retinal artery (CRA) is located in the center of the optic nerve and accompanied by the central retinal vein (CRV) and that the vessels are surrounded by nerve fibers. The diameter of the central retinal artery is 250 μm. Hematoxylin–eosin stain, scale bars = 200 μm

The ophthalmic artery system was isolated completely through the dissection of connective tissues and was shown to be specifically suited to nourish the eyeball. The central retinal artery was the only artery that inserted vertically into the optic nerve at a distance of 0.9 ± 0.22 cm from the eyeball, and the posterior ciliary arteries had several collateral circulations (Fig. 2). The central retinal artery twines around the inferior aspect of the optic nerve, and its diameter was approximately 250 μm in one cadaver specimen (Fig. 3).

## Discussion

**Fig. 4:**
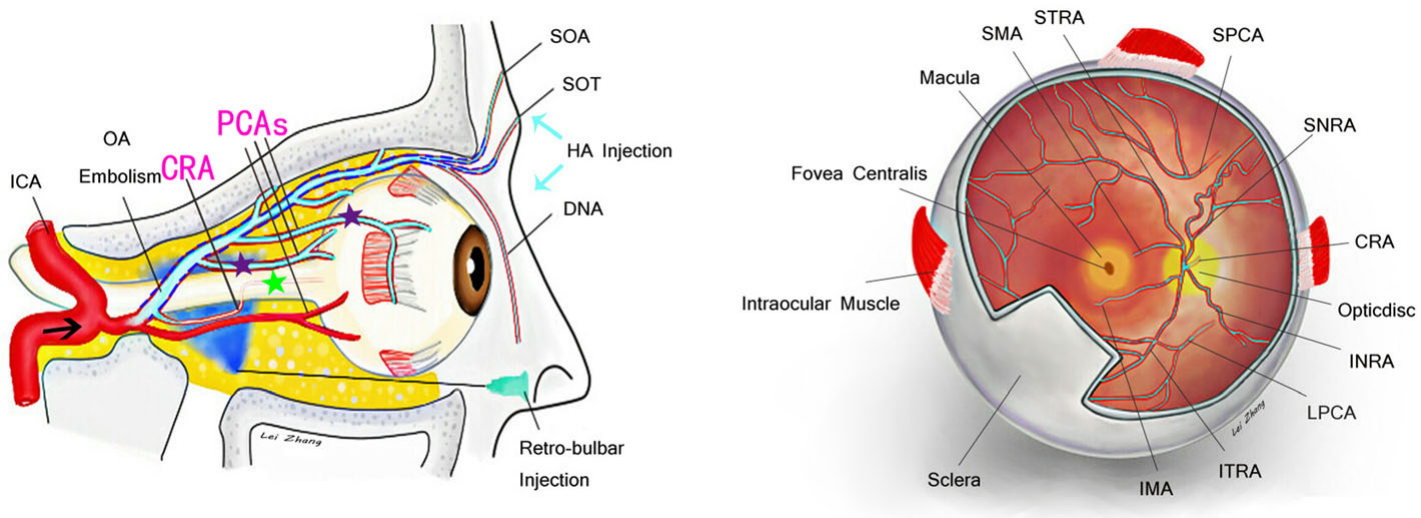
Schematic diagrams of ophthalmic artery embolism. The ophthalmic artery arising from the internal carotid artery (ICA) has facial branches, including the supraorbital artery (SOA), supratrochlear artery (STA) and dorsal nasal artery (DNA), and intraocular branches, including the central retinal artery (CRA) and the posterior ciliary arteries (PCAs). The CRA is the only blood vessel that nourishes the entoretina, and it has four branches, including the superior temporal retinal artery (STRA), the superior nasal retinal artery (SNRA), the inferior temporal retinal artery (ITRA) and the inferior nasal retinal artery (INRA). PCAs, including the short posterior ciliary artery (SPCA) and the long posterior ciliary artery (LPCA), insert into the sclera to nourish the ectoretina. The macula is nourished by the superior macular arteriole (SMA) and the inferior macular arteriole (IMA). Embolism of these arteries (stars) caused by a facial area injections (arrows) could lead to different visual impairment in the corresponding areas. The arterial volumes found in cadavers (dash line) were calculated in our study. The tip of the sharp needle indicated the site of retrobulbar hyaluronidase injection (blue color)

The arrangement of arterial vessels supplying the eyeball is unusual as anastomotic communications are present between the external carotid artery system (the facial artery and its branches) and the internal carotid artery system (supraorbital, supratrochlear and dorsal nasal arteries). This opens up possibilities that filler injections anywhere in the territory of the facial artery could retrogradely be injected into the ophthalmic artery to occlude it. The ophthalmic artery, which arises from the internal carotid artery and gives off intraocular branches and facial branches, is the primary blood vessel for the retina and adnexa oculi. Ophthalmic artery embolism occurs when fillers are inadvertently injected into facial branches, including the supraorbital artery, supratrochlear artery and dorsal nasal artery, with backflow of the filler material into the trunk of the ophthalmic artery against its natural direction of flow [9, 17]. With strong arterial perfusion, an HA embolus causing contraflow can move forward and occlude the intraocular branches of the ophthalmic artery, including the central retinal artery and posterior ciliary arteries, eventually causing ocular symptoms such as ptosis, strabismus and even blindness (Fig. 4). The further the backflow of this column of filler material along the ophthalmic artery to its origin is, the more severe the degree of blindness or visual impairment will be.

The extent of visual impairment from partial to complete visual field loss depends therefore on the location of the HA embolism. The central retinal artery runs into the optic disk and has four branches, including the superior temporal retinal artery, the superior nasal retinal artery, the inferior temporal retinal artery and the inferior nasal retinal artery. The central retinal artery is the only blood vessel that nourishes the entoretina, while the short posterior ciliary artery and long posterior ciliary artery insert into the sclera to nourish the ectoretina (Fig. 4). A type I embolism of the central retinal artery usually causes complete visual field loss, such as that observed in Patient No. 1. A type II embolism of the posterior ciliary artery usually causes partial visual field impairment due to collateral circulation, as observed in Patient No. 2. A type III embolism involving double embolism of the central retinal artery and posterior ciliary artery is the most severe and usually causes complete visual field loss, such as that observed in Patient No. 3. The macula is nourished by the superior macular arteriole and the inferior macular arteriole, the embolism of which may also cause serious visual impairment. Therefore, the severity of visual impairment caused by HA injection might be associated with the occlusion site, and visual impairment is more severe in central retinal artery occlusion and combined intraocular branch occlusion than in posterior ciliary artery occlusion.

Visual impairment caused by HA injection is catastrophic, and there is no widely recognized effective treatment for this complication [23]. Carruthers et al. [18] proposed retrobulbar injection of hyaluronidase to reverse visual impairment after filler injection. It remains possible that extravascular hyaluronidase can diffuse through the ophthalmic artery or its branches to dissolve an intravascular HA embolus. Zhu et al. [20] reported that retrobulbar injection of hyaluronidase was unable to recanalize occluded retinal arteries or improve the visual outcome in four patients with visual impairment caused by facial HA injection. We reviewed the cases of three patients suffering from visual impairment and ischemic neuropathy after HA injection. Two of these patients had no visual improvement after retrobulbar injection. We believe that the specific anatomic characteristics of the ophthalmic artery and the limited ischemic tolerance of the retina may lead to greater difficulty in relieving visual impairment than that experience following cutaneous necrosis caused by HA injection.

To date, one successful rescue of visual impairment after retrobulbar injection of hyaluronidase has been reported [24]. Ocular angiography and ophthalmic testing were not performed in this patient, and the exact site of embolism was therefore not clear. This patient was also injected with hyaluronidase into the infraorbital foramen and supraorbital notch. Another patient with visual impairment was reported to have recovered after the injection of hyaluronidase into the supraorbital notches [25]. Therefore, Goodman suggested that hyaluronidase might need to be directly injected into a vessel through the supraorbital notch [26]. Intravascular administration of hyaluronidase via endovascular intervention may be another potential approach to reversing vision loss [26, 27]. In the published case, partial recanalization of the ophthalmic artery and its branches was achieved, and ocular motility was restored, but the visual outcome was not improved by this treatment because of the limited ischemic tolerance of the retina [27].

A thorough understanding of anatomy and injection techniques is vital to prevent this complication and it is advisable for all physicians who perform facial filler injections to receive appropriate anatomical teaching [28–30]. Our previous anatomy study showed that deep injections in the periosteal plane or sub-SMAS plane are not advisable in the glabellar region and nasal dorsum [9]. In this study, the minimum dose of HA that could cause central retinal artery embolism was calculated to be as little as 0.08 ml. Therefore, limiting the volume of each droplet per injection may be an important consideration in reducing the risk of visual impairment, especially for injections in the glabellar region and nasal dorsum.

A limitation of this research is that it is difficult to accurately estimate the dose of HA that can lead to ophthalmic artery embolism, causing visual impairment during facial HA injection based on cadaver anatomical measurements of arterial volumes. In addition, the symptoms of these patients varied; more cases of facial filler-related visual impairment should be studied and reported to enhance our understanding of the optimal treatment and prevention strategies for this catastrophic complication.

## Conclusions

The extent of blindness or visual impairment due to embolism depended on the site of the embolism in the intraocular branches of the ophthalmic artery, and no cases showed any improvements in vision after treatment. As little as 0.08 ml of HA can cause central retinal artery embolism following facial branch injection. Limiting the volume of each droplet per injection is a simple technique to potentially reduce this complication.

## References

[1] Hedén P, Sellman G, Wachenfeldt MV (2009). Body shaping and volume restoration: the role of hyaluronic acid. Aesthet Plast Surg.

[2] Gottfried L, Rullan Peter P, Nelly G-H (2006). Avoiding and treating dermal filler complications. Plast Reconstr Surg.

[3] Tae-Hwan P, Sang-Won S, June-Kyu K, Choong-Hyun C (2011). Clinical experience with hyaluronic acid-filler complications. J Plast Reconstr Aesthet Surg.

[4] Kim DY, Eom JS, Kim JY (2015). Temporary blindness after an anterior chamber cosmetic filler injection. Aesthet Plast Surg.

[5] Liew S, Wu WTL, Chan HH (2016). Consensus on changing trends, attitudes, and concepts of asian beauty. Aesthet Plast Surg.

[6] Lazzeri D, Agostini T, Figus M (2015). Blindness following cosmetic injections of the face. Plast Reconstr Surg.

[7] Hayreh SS, Weingeist TA (1980). Experimental occlusion of the central artery of the retina. IV: Retinal tolerance time to acute ischaemia. Br J Ophthalmol.

[8] Hayreh SS, Podhajsky PA, Zimmerman B (1997). Nonarteritic anterior ischemic optic neuropathy: time of onset of visual loss. Am J Ophthalmol.

[9] Wu S, Pan L, Wu H (2017). Anatomic study of ophthalmic artery embolism following cosmetic injection. J Craniofac Surg.

[10] Goodman GJ, Roberts S, Callan P (2016). Experience and management of intravascular injection with facial fillers: results of a multinational survey of experienced injectors. Aesthet Plast Surg.

[11] Kim YS, Choi DY, Gil YC (2015). The anatomical origin and course of the angular artery regarding its clinical implications. Dermatol Surg.

[12] Lang J, Kageyama I (1990). The ophthalmic artery and its branches, measurements and clinical importance. Surg Radiol Anat.

[13] Hayreh SS (2006). Orbital vascular anatomy. Orbit.

[14] Perrini P, Cardia A, Fraser K (2007). A microsurgical study of the anatomy and course of the ophthalmic artery and its possibly dangerous anastomoses. J Neurosurg.

[15] Khan TT, Colonacevedo B, Mettu P (2017). An anatomical analysis of the supratrochlear artery: considerations in facial filler injections and preventing visual impairment. Aesthet Surg J.

[16] Scheuer JF, Sieber DA, Pezeshk RA (2017). Anatomy of the facial danger zones: maximizing safety during soft-tissue filler injections. Plast Reconstr Surg.

[17] Carruthers JD, Fagien S, Rohrich RJ (2015). Blindness caused by cosmetic filler injection: a review of cause and therapy. Plast Reconstr Surg.

[18] Carruthers J, Fagien S, Dolman P (2015). Retro or peribulbar injection techniques to reverse visual loss after filler injections. Dermatol Surg.

[19] Hwang CJ, Mustak H, Gupta AA (2018). Role of retrobulbar hyaluronidase in filler-associated blindness: evaluation of fundus perfusion and electroretinogram readings in an animal model. Ophthalm Plast Reconstr Surg.

[20] Zhu GZ, Sun ZS, Liao WX (2015). Efficacy of retrobulbar hyaluronidase injection for visual impairment resulting from hyaluronic acid filler embolization. Aesthet Surg J.

[21] Loh KTD, Chua JJ, Lee HM (2015). Prevention and management of visual impairment relating to facial filler injections. Singap Med J.

[22] Signorini M, Liew S, Sundaram H (2016). Global aesthetics consensus: avoidance and management of complications from hyaluronic acid fillers-evidence and opinion-based review and consensus recommendations. Plast Reconstr Surg.

[23] Carle MV, Roe R, Novack R (2014). Cosmetic facial fillers and severe visual impairment. JAMA Ophthalmol.

[24] Chesnut C (2018). Restoration of visual loss with retrobulbar hyaluronidase injection after hyaluronic acid filler. Dermatol Surg.

[25] Goodman GJ, Clague MD (2016). A rethink on hyaluronidase injection, intraarterial injection, and blindness: Is there another option for treatment of retinal artery embolism caused by intraarterial injection of hyaluronic acid?. Dermatol Surg.

[26] Foissac R, Kestemont P, Camuzard O (2016). Intravenous hyaluronidase with urokinase as treatment for arterial hyaluronic acid embolism. Plast Reconstr Surg.

[27] Oh BL, Jung C, Park KH, Hong YJ, Woo SJ (2014). Therapeutic intra-arterial hyaluronidase infusion for ophthalmic artery occlusion following cosmetic facial filler (hyaluronic acid) injection. Neuro-Ophthalmology.

[28] Katie B, Carruthers Jean DA, Shannon H, Derek J (2015). Avoiding and treating blindness from fillers: a review of the world literature. Dermatol Surg.

[29] Narendra K, Eqram R (2017). Effectiveness of teaching facial anatomy through cadaver dissection on aesthetic physicians’ knowledge. Adv Med Educ Pract.

[30] Narendra K, Eqram R, Adds Philip J (2018). An effective and novel method for teaching applied facial anatomy and related procedural skills to esthetic physicians. Adv Med Educ Pract.

